# Mir-184 Post-Transcriptionally Regulates SOX7 Expression and Promotes Cell Proliferation in Human Hepatocellular Carcinoma

**DOI:** 10.1371/journal.pone.0088796

**Published:** 2014-02-18

**Authors:** Geng-Gang Wu, Wen-Hong Li, Wen-Guang He, Nan Jiang, Guang-Xian Zhang, Wei Chen, Hai-Feng Yang, Qi-Long Liu, Yan-Nian Huang, Lei Zhang, Tong Zhang, Xian-Cheng Zeng

**Affiliations:** 1 Department of General Surgery, Zengcheng People’s Hospital (BoJi-Affiliated Hospital of Sun Yat-Sen University), Zengcheng, China; 2 Liver Transplantation Center, Third Affiliated Hospital, Sun Yat-sen University, Guangzhou, China; 3 School of Basic Medical Sciences, Guangzhou University of Chinese Medicine, Guangzhou, China; 4 Department of Hepatopancreatobiliary Surgery, Second Affiliated Hospital, School of Medicine, Zhejiang University, Hangzhou, China; 5 Department of Pathology, The Second Affiliated Hospital of Guangzhou University of Chinese Medicine (Guangdong Provincial Hospital of TCM), Guangzhou, China; 6 Department of Clinical Laboratory, Zengcheng People’s Hospital (BoJi-Affiliated Hospital of Sun Yat-Sen University), Zengcheng, China; University of Modena & Reggio Emilia, Italy

## Abstract

Hepatocellular carcinoma (HCC) is one of the most common human malignancies and the third leading cause of cancer mortality worldwide. The development and progression of HCC is a complicated process, involving the deregulation of multiple genes that are essential to cell biological processes. Recently, microRNAs (miRNAs) have been suggested to be closely associated with tumorigenesis. Our study showed that miR-184 is upregulated in HCC cell lines and tissues. Overexpression of miR-184 in HCC cells increased cell proliferation, tumorigenicity, and cell cycle progression, whereas inhibition of miR-184 reduced cell proliferation, tumorigenicity, and cell cycle progression. Additionally, we identified SOX7 as a direct target of miR-184. Ectopic expression of miR-184 led to downregulation of the SOX7 protein, resulting in upregulation of c-Myc, Cyclin D1, and phosphorylation of Rb. Our findings suggested that miR-184 represents a potential onco-miR and plays an important role in HCC progression by suppressing SOX7 expression.

## Introduction

Hepatocellular carcinoma (HCC) is the fifth most common cancer and the third most common cause of cancer mortality in the world [1], [2]. Due to the high morbidity of hepatitis B virus (HBV) and hepatitis C virus (HCV) infection, the incidence of HCC is higher in Asian countries. The HCC incidence is much higher in China than in other Asian countries, and Chinese cases account for 55% of all HCC cases worldwide [2]. The absence of routine screening means that most HCC cases in China are initially diagnosed at advanced stages, and patients die from tumor growth or liver failure [3]. The late onset of clinical symptoms accounts for the late diagnosis and poor prognosis; the identification of reliable biomarkers for HCC is especially important [4]. Moreover, further studies on the biology involved in HCC initiation and progression are also urgently needed to develop effective diagnostic methods and therapeutic strategies.

The discovery of miRNAs and their function in tumor progression provide new sights for HCC research. MiRNAs are a class of endogenous, small non-coding RNAs of 20–22 nucleotides that involved in multiple biological processes [5], [6]. MiRNAs have been identified as a new type of gene expression regulators, which negatively regulate gene expression at the post-transcriptional level by targeting the 3′ untranslated region (3′-UTR) of mRNAs in a sequence-specific manner [7], [8]. Recent studies showed that miRNAs play essential roles in the biology of various human cancers, including cell differentiation, proliferation, apoptosis, invasion and angiogenesis [9], [10]. Based on the close relationship between miRNAs and carcinogenesis, miRNAs are presently considered as potential targets for caner diagnosis and anti-cancer therapies [11]–[13].

As a member of the F subfamily of the SRY-related high mobility group box (SOX) transcription factors, the sex determining region Y (SRY)-box 7 (SOX7) protein is one of tumor-suppressive genes regulated by some onco-microRNAs. SOX7 mediates various biological processes, including the regulation of hematopoiesis, vasculogenesis, cardiogenesis, endoderm differentiation and myogenesis [14]–[18]. Furthermore, SOX7 functions as a tumor suppressor in lung cancers, endometrial cancer, colorectal cancer, prostate cancer and breast cancer [19]–[24]. However, the function of SOX7 in HCC progression has not been determined.

In this study, we showed that miR-184 is upregulated in HCC cell lines and tissues. We further found that ectopic expression of miR-184 in HCC cells led to the promotion of cell proliferation, tumorigenicity and cell cycle regulation. Moreover, we presented data indicating that SOX7 is a direct target of, and is downregulated by, miR-184. Thus, miR-184 plays essential role during the regulation of SOX7 in HCC cells *in vitro*. Our present study suggested that miR-184 promotes cell proliferation, tumorigenicity and cell cycle progression in HCC cells by targeting SOX7 mRNA and suppressing its expression.

## Materials and Methods

### Cell Culture

Immortalized normal liver epithelial cells, THLE3, were from the American Type Culture Collection (ATCC, Manassas, VA, USA) and were cultured under the conditions stated by the manufacturer. The HCC cell lines (Hep3B, BEL-7402, MHCC97H, HCCC-9810, MHCC97L, Huh7, QGY-7703 and HepG2, purchased from ATCC) were grown in Dulbecco’s modified Eagle’s medium (DMEM, Invitrogen, Carlsbad, CA, USA) supplemented with 10% fetal bovine serum (FBS, Invitrogen), at 37°C in a 5% CO_2_ atmosphere in a humidified incubator.

### Tissue Specimens

All experimental procedures were carried out in strict accordance with institutional guidelines and human tissue samples were obtained according to protocols approved by the Third Affiliated Hospital of Sun Yat-sen University ethics Committee. For the use of clinical materials for research purposes, samples were obtained with prior written informed consents from the patients and approval from the Institutional Research Ethics Committees of Third Affiliated Hospital of Sun Yat-sen University ethics Committee. This study was conducted on eight pairs of snap-frozen HCC tumors and matched normal tissues from adjacent regions, which were diagnosed histopathologically at Third Affiliated Hospital of Sun Yat-sen University from 2001 to 2006. The eight HCC tissues (stage I: No. 1, 7; stage II: No. 3, 4, 8; stage III: No.2, 5, 6) and the matched adjacent noncancerous tissues, which had been histopathologically diagnosed and verified by experienced pathologists, were frozen and stored in liquid nitrogen until further use.

### Generation of Stably Engineered Cell Lines

pMSCV-miR-184, the miR-184 expression plasmid, was generated by cloning the genomic pre-miR-184 gene with 300-bp on each flanking side into the retroviral transfer plasmid pMSCV-puro (Clontech Laboratories Inc., Mountain View, CA, USA). pMSCV-miR-184 was then cotransfected with the pIK packaging plasmid into 293FT cells, using the standard calcium phosphate transfection method [25]. Thirty-six hours after cotransfection, supernatants were collected and incubated with HCC cells to be infected for 24 hours in the presence of polybrene (2.5 µg/ml, Sigma, Saint Louis, MO, USA). After infection, puromycin (1.5 µg/ml, Sigma) was used to select stably transduced cells over 10 days.

### RNA Extraction and Real-time Quantitative PCR

Total cellular RNA was extracted using the Trizol reagent (Invitrogen), according to the manufacturer’s instruction. cDNAs were synthesized and real-time PCR was performed using the GoTaq® 2-Step RT-qPCR System (Promega, Madison, WI, USA) in an ABI Prism 7500 Sequence Detection System (Applied Biosystems, Foster City, CA, USA). Expression levels of genes were normalized to that of the housekeeping gene *GAPDH* as the control and calculated as 2^−[(Ct^ ^of *MYC*, *CyclinD1*) – (Ct^ ^of *GAPDH*)]^, where C_t_ represents the threshold cycle for each transcript. The expression of the miRNA was defined based on C_t_, and relative expression levels were calculated as 2^−[(Ct of miR−184) – (Ct of U6)]^ after normalization with reference to the expression of small nuclear RNA U6. The extracted RNA was pretreated with RNase-free DNase, and 500ng of RNA from each sample was used for cDNA synthesis primed with the specific microRNA RT-primer purchased from RiboBio (RiboBio Co. Ltd, Guangzhou, Guangdong, China). For PCR amplification of cDNA, the primers of *miR-184* and *U6* were purchased from RiboBio, and an initial amplification using primers was done with a denaturation step at 95°C for 20 seconds, followed by 40 cycles of denaturation at 95°C for 10 seconds, primer annealing at 60°C for 20 seconds, and primer extension at 70°C for 5 seconds.

The primers used were:

*MYC* forward: 5′- TCAAGAGGCGAACACACAAC -3′,

*MYC* reverse: 5′- GGCCTTTTCATTGTTTTCCA -3′;

*Cyclin D1* forward: 5′- AACTACCTGGACCGCTTCCT -3′,

*Cyclin D1* reverse: 5′- CCACTT GAGCTTGTTCACCA-3′;

*LEF1* forward: 5′- CACTGTAAGTGATGAGGGGG -3′;

*LEF1* reverse: 5′- TGGATCTCTTTCTCCACCCA -3′;

*TCF* forward: 5′- GTGTACTTGGATGAGTTTCGTCG -3′;

*TCF* reverse: 5′- TTGCCAC ATTAAAGGCAGCTC -3′;

*GAPDH* forward: 5′- GACTCATGACCACAGTCCATGC -3′,

*GAPDH* reverse: 3′- AGAGGCAGGGATGATGTTCTG -5′.

### Western Blotting

Western blotting was performed according to a previously reported method [26]. The membranes were probed with polyclonal rabbit antibodies, anti-SOX7 (#ab80331, 1∶500; Abcam, Cambridge, MA, USA), anti-c-Myc (#9402), anti-CyclinD1 (#2978), anti-Rb (#9313) and anti-phosphorylated Rb (#9306; 1∶1,000; Cell Signaling, Danvers, MA, USA). The membranes were then stripped and re-probed with an anti-α-Tubulin mouse monoclonal antibody (#2125; 1∶1,000; Cell Signaling) as a loading control.

### Plasmids, Oligonucleotides, siRNA and Transfection

The region of the human SOX7 3′-UTR, generated by PCR amplification from DNA of HepG2 cells, was cloned into vector pGL3 (Promega). The primers used were:

SOX7-3′UTR-wt forward: 5′-GCCCCGCGGGCCCTCTCCTTCTTGTGCCTTG-3′;

SOX7-3′UTR-wt reverse: 3′-GCCCTGCAGGGATAGAGGCGGCACTCGGATAA-5′.

SOX7-3′UTR-mut forward: 5′-GCCTTGAGTGGCAGAGGAGCCGCAAAGCCACACCAGCTTTCCTCCC-3′; SOX7-3′UTR-mut reverse:

5′-GGGAGGAAAGCTGGTGTGGCTTTGCGGCTCCT CTGCCACTCAAGGC-3′.

The miR-184 mimic, miR-184 inhibitor (miR-184 inhibitor is a LNA/OMe modified antisense oligonucleotide designed specifically to bind to and inhibit endogenous miR-184 molecule) and negative control were purchased from RiboBio. For depletion of SOX7, two siRNAs were synthesized and purified by RiboBio (SOX7 siRNA#1 sequence: CCACTCCACTCCAACCTCCAA, SOX7 siRNA#2 sequence: GCCGAGCTGTCGGATGGACAA). Transfection of oligonucleotides and siRNA were performed using the Lipofectamine 2000 reagent (Invitrogen), according to the manufacturer’s instruction.

### Luciferase Assay

Cells were seeded in triplicate in 24-well plate and allowed to settle for 24 h. One hundred nanograms of pGL3-SOX7-luciferase plasmid (or Mut) was transfected into HCC cells using the Lipofectamine 2000 reagent, according to the manufacturer’s instruction. Luciferase and control signals were measured at 48 h after transfection using the Dual Luciferase Reporter Assay Kit (Promega), according to a protocol provided by the manufacturer. Three independent experiments were performed and the data were presented as the mean ± SD.

3-(4, 5-Dimethyl-2-thiazolyl)-2, 5-diphenyl-2H-tetrazolium bromide (MTT) assay.

Cells were seeded on 96-well plates and stained at indicated time points with 100 µl sterile MTT dye (0.5 mg/ml, Invitrogen) for 4 h at 37°C, followed by removal of the culture medium and addition of 150 µl of dimethyl sulfoxide (DMSO) (Sigma). The absorbance was measured at 570 nm, with 655 nm as the reference wavelength. All experiments were performed in triplicate.

### Colony Formation Assay

Cells were plated on a 6-well plate (1×10^3^ cells per well) and cultured for 10 days. The colonies were stained with 1.0% crystal violet for 5 min after fixation with 10% formaldehyde for 15 min. All experiments were performed in triplicate.

### Anchorage-independent Growth Ability Assay

Cells (1×10^3^) were trypsinized and suspended in 2 ml complete medium plus 0.33% agar (Invitrogen). The mixture was plated on top of a bottom layer comprising 0.66% complete medium-agar mixture on a 6-well plate. After 10 days incubation, colony sizes were measured with an ocular micrometer and colonies greater than 0.1 mm in diameter were scored. All experiments were performed in triplicate.

### Flow Cytometry Analysis

All cells in a culture dish were harvested by trypsinization, washed in ice-cold PBS, and fixed in 80% ice-cold ethanol in PBS. The cells were then pelleted and resuspended in cold PBS. Bovine pancreatic RNAase (2 µg/ml, Sigma) was added and cells were incubated at 37°C for 30 min, followed by incubation in propidium iodide (10 µg/ml, Invitrogen) for 30 min at room temperature. Twenty thousand cells were analyzed by flow cytometry (FACSCalibur; BD Biosciences, San Jose, CA, USA). All experiments were performed in triplicates.

### Statistical Analysis

Student’s *t* test was used to evaluate the significant difference between two groups of data in all the pertinent experiments. The experimental data were represented from three biological independent replicates and as the mean ± SD. A *P* value <0.05 (using a two-tailed paired *t* test) was considered statistically significant.

## Results

### MiR-184 Expression is Elevated in HCC

**Figure 1:**
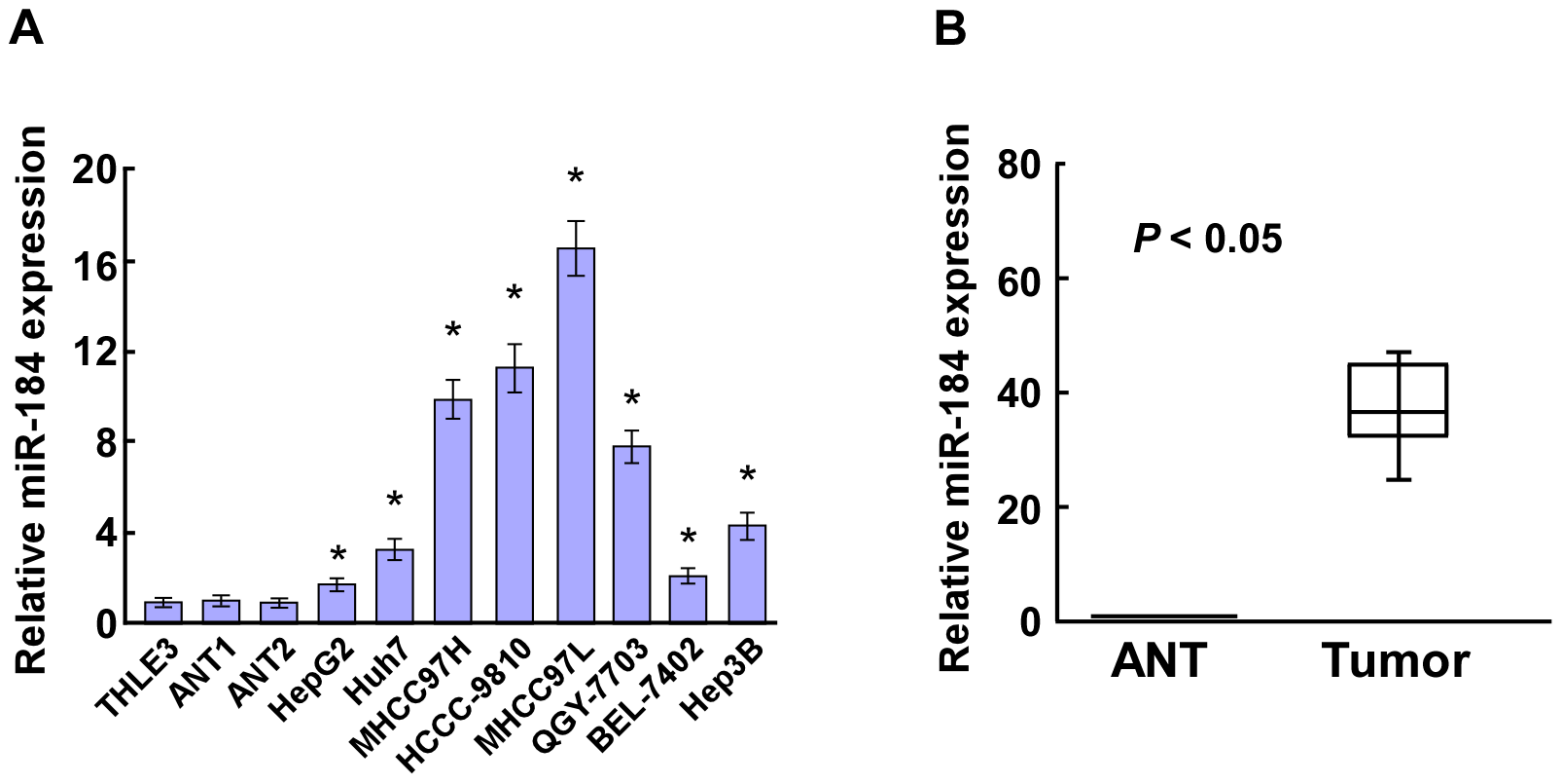
Expression of miR-184 is elevated in HCC. **A**. Real-time PCR analysis of miR-184 expression in hepatocellular carcinoma cell lines (Hep3B, BEL-7402, MHCC97H, HCCC-9810, MHCC97L, Huh7, QGY-7703 and HepG2), compared with normal liver epithelial THLE3 cells and two noncancerous hepatic tissues (ANT1 and ANT2). **B**. The expression of miR-184 was examined in eight paired cancerous tissues (T) and their adjacent noncancerous hepatic tissues (ANT), showed in a boxplot. The average miR-184 expression was normalized using U6 expression. Each bar represents the mean ± SD of three independent experiments. *P<0.05.

By analyzing a published micro-array-based high-throughput assessment (NCBI/GEO/GSE31384), miR-184 was identified to be significantly upregulated in HCC tissues compared with that in matched noncancerous hepatic tissues. Real-time PCR analysis showed that miR-184 expression was markedly increased in all eight HCC cell lines (Hep3B, BEL-7402, MHCC97H, HCCC-9810, MHCC97L, Huh7, QGY-7703 and HepG2), compared with that in the normal liver epithelial cells THLE3 and the other two noncancerous hepatic tissues (Figure 1A). Comparative analysis also revealed that miR-184 was significantly upregulated in eight pairs of cancerous tissues compared with the adjacent noncancerous hepatic tissues (Figure 1B, Figure S1). Collectively, our results showed that miR-184 was overexpressed in HCC cell lines and tissues.

### Ectopic Expression of miR-184 Enhances Proliferation of HCC Cells

**Figure 2:**
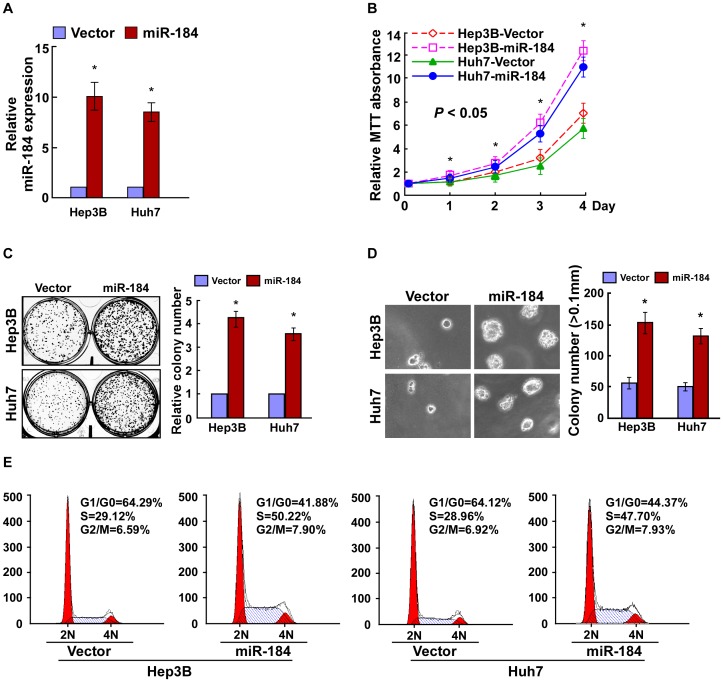
Ectopic miR-184 promotes the proliferation of HCC cells. **A**. Real-time PCR analysis of miR-184 expression in Hep3B and Huh7 cells stably overexpressing miR-184. **B**. Effects of ectopic miR-184 on the proliferation of the indicated HCC cell lines, as analyzed by the MTT assay. **C**. Representative micrographs (left) and quantifications (right) of crystal violet stained cell colonies formed by the indicated HCC cell lines, 10 days after inoculation. **D**. Effects of ectopic miR-184 on the tumorigenicity of the indicated HCC cell lines, as determined by anchorage-independent growth ability assay. Colonies larger than 0.1 mm were scored. **E**. Effects of ectopic miR-184 on the cell cycle progression of the indicated HCC cells, as measured by flow cytometry analysis. Each bar represents the mean ± SD of three independent experiments. *P<0.05.

To determine the effect of miR-184 on HCC progression, Hep3B and Huh7 cells stably overexpressing miR-184 were established for further study (Figure 2A). An MTT assay showed that ectopic expression of miR-184 significantly increased the growth rate of Hep3B and Huh7 cells (Figure 2B). Colony formation assays showed a similar result: overexpression of miR-184 enhanced the proliferation of HCC cells (Figure 2C). Additionally, an anchorage-independent growth assay revealed that Hep3B and Huh7 cells stably expressing miR-184 showed more and larger-sized colonies than control cells (Figure 2D). We further analyzed the cell cycle of Hep3B and Huh7 cells by flow cytometry. The results showed a dramatic decrease in the percentage of cells in G1/G0 phase and an increase in the percentage of cells in S phase (Figure 2E). The results suggested that upregulation of miR-184 promoted the proliferation and tumorigenicity of HCC cells *in vitro*.

### Inhibition of miR-184 Reduces Proliferation of HCC Cells

**Figure 3:**
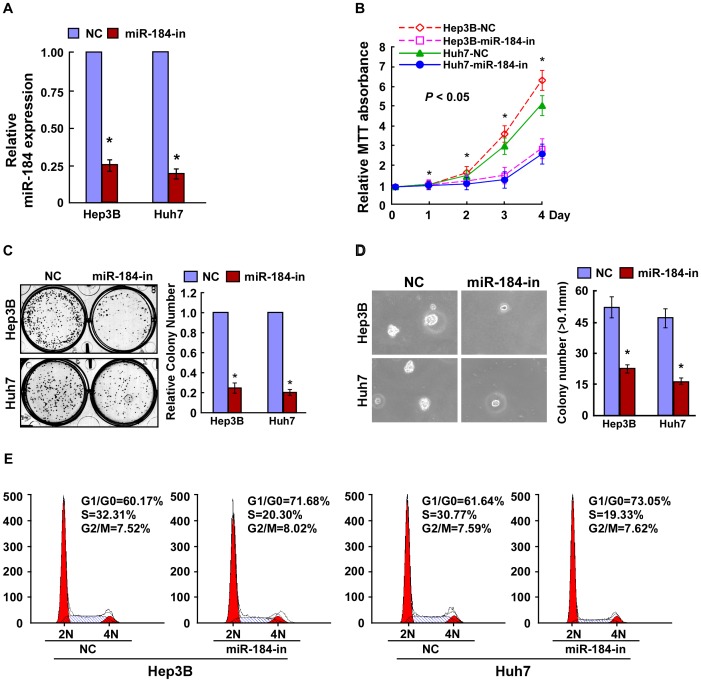
Inhibition of miR-184 reduces the proliferation of HCC cells. **A**. Real-time PCR analysis of miR-184 expression in Hep3B and Huh7 cells transfected with miR-184 inhibitor. **B**. The proliferation ability of HCC cells transfected with an miR-184 inhibitor or negative control (NC), analyzed by the MTT assay. **C**. Representative micrographs (left) and quantifications (right) of crystal violet stained cell colonies formed by indicated HCC cell lines, 10 days after inoculation. **D**. The tumorigenicity of HCC cells transfected with miR-184 inhibitor or NC, as determined by an anchorage-independent growth ability assay. Colonies larger than 0.1 mm were scored. **E**. Flow cytometry analysis of indicated HCC cells transfected with miR-184-inhibitor or NC. Each bar represents the mean ± SD of three independent experiments. *P<0.05.

To further test whether endogenous miR-184 helps to sustain the proliferative property of HCC cells, loss-of-function studies using a miR-184 inhibitor were performed (Figure 3A). The results showed that suppression of miR-184 significantly decreased the growth rate of Hep3B, Huh7 and THLE3, when transfected with the miR-184 inhibitor, compared with that of NC transfected cells (Figure 3, B and C, and Figure S2A). The anchorage-independent growth assay revealed that Hep3B-miR-184-inhibitor and Huh7-miR-184-inhibitor cells produced fewer and smaller colonies than the negative control cells, indicating the inhibitory function of the miR-184 inhibitor on HCC tumorigenicity (Figure 3D). In addition, flow cytometry showed a significant increase in the percentage of cells in G1/G0 phase and a decrease in the percentage of cells in S phase in cells transfected with the miR-184 inhibitor compared with NC transfected cells (Figure 3E). These results suggested that downregulation of miR-184 could reduce the proliferation and tumorigenicity of HCC cells.

### SOX7 is a Direct Target of miR-184 in HCC Cells

**Figure 4:**
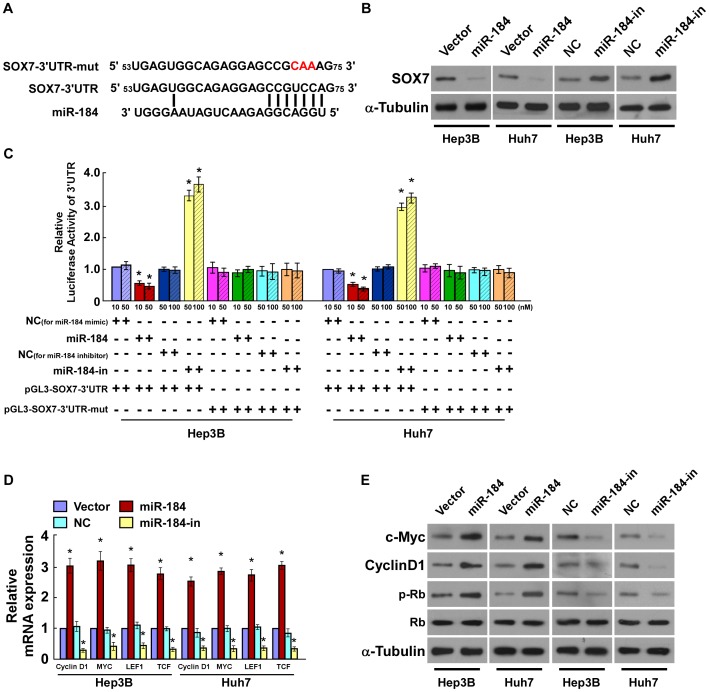
MiR-184 directly targets the 3′-UTR of *SOX7* mRNA. **A**. Schematic representation of the mature miR-184 sequence, miR-184 target site in the 3′-UTR of *SOX7* mRNA and a 3′-UTR mutant of *SOX7* mRNA containing three altered nucleotides in the putative target site (SOX7-3′UTR-mut). **B**. The expression levels of SOX7 protein in HCC cells overexpressing miR-184 or transfected with miR-184 inhibitor, compared with control cells, by western blotting 48 hours after transfection; α-Tubulin served as the loading control. **C**. Luciferase assay of pGL3-SOX7-3′UTR or pGL3-SOX7-3′UTR-mut reporter cotransfected with different amounts (10, 50 nM) of miR-184 mimic in indicated cells, or different amounts (50, 100 nM) of miR-184 inhibitor, compared with negative control (NC). **D**. Real-time PCR analysis of the mRNA expression of genes, *MYC*, *CyclinD1, LEF1* and *TCF*, in indicated HCC cells. **E**. Expression of c-Myc, CyclinD1, phosphorylated Rb, and Rb protein, as measured by western blotting in indicated HCC cells; α-Tubulin served as the loading control. Each bar represents the mean ± SD of three independent experiments. *P<0.05.

To explore the molecular mechanism of miR-184 function in HCC cells, we used publicly available algorithms (TargetScan, Pictar and miRANDA, which are public compilation of databases and web portals and servers used for microRNAs and their targets) to predict the target(s) of miR-184 in humans. The results indicated that SOX7 was one of the potential targets of miR-184 (Figure 4A). SOX7 was previously reported as a tumor suppressor in various cancers [19]–[23]. As predicted, western blotting revealed that SOX7 expression decreased in HepG3 and Huh7 cells, compared with normal cells THLE3, and further decreased in HepG3 and Huh7 cells overexpressing miR-184 and increased in cells transfected with the miR-184 inhibitor (Figure 4B, Figure S2B). To examine whether miR-184 mediated-SOX7 downregulation was effected via the 3′-UTR of SOX7, the SOX7-3′-UTR fragment, containing miR-184 binding site, was subcloned into a pGL3 luciferase reporter vector. The results of the luciferase reporter assay showed that ectopic expression of miR-184 decreased, and suppression of miR-184 increased, the luciferase activity of the SOX7 3′-UTR-luciferase reporter. By contrast, a SOX7 3′-UTR -luciferase reporter with a mutant miR-184 binding site seed sequence was not inhibited by ectopic expression of miR-184 (Figure 4C). Considering it was previously reported that SOX7 could remarkably reduce Wnt/β-catenin signaling activity and the expression of its downstream genes [21], [27], [28], we further examined the expression of some Wnt/β-catenin signaling related genes, *Cyclin D1, MYC*, *LEF1,* and *TCF.* The results showed that the mRNA of *Cyclin D1, MYC*, *LEF1,* and *TCF* were significantly upregulated by ectopic miR-184, whereas they were downregulated by inhibition of miR-184 (Figure 4D). Moreover, the expression of c-Myc and Cyclin D1 proteins were upregulated, and phosphorylated Rb was increased in miR-184 overexpressing cells compared with the negative control cells (Figure 4E). By contrast, the expression of c-Myc and Cyclin D1 were downregulated, and Rb phosphorylation was decreased, in cells transfected with the miR-184 inhibitor (Figure 4E). Furthermore, we examined the expression level of these Wnt/β-catenin signaling related genes in HCC tissues. The result showed that *Cyclin D1, MYC*, and *LEF1* were upregulated in HCC tissues and the expression levels were positively correlated with the expression of miR-184 (Figure S3). Collectively, our results suggested that SOX7 is a direct target of miR-184.

### SOX7 Suppression is Critical for miR-184-induced Cell Proliferation in HCC

**Figure 5:**
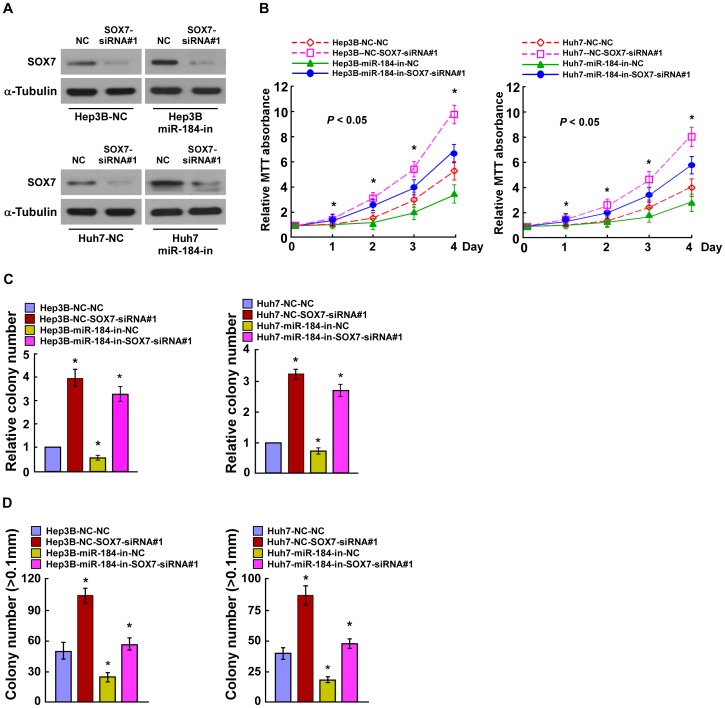
MiR-184 promotes HCC proliferation by inhibiting SOX7. **A**. The expression levels of SOX7 in miR-184-inhibitor transfected HCC cells that were transfected with SOX7-siRNA#1, as measured by western blotting; α-Tubulin served as the loading control. **B**. The growth rates in SOX7-silenced cells, as indicated by the MTT assay. **C**. Quantifications of crystal violet stained cell colonies formed by indicated HCC cell lines, 10 days after inoculation. **D**. Quantifications of colony numbers of indicated cells determined by an anchorage-independent growth assay. Colonies larger than 0.1 mm in diameter were scored. Error bars represent the mean ± SD from three independent experiments. *P<0.05.

To evaluate the effect of SOX7 suppression on HCC progression, we suppressed endogenous SOX7 expression with two SOX7-specific siRNAs (Figure 5A, Figure S4A). The MTT and the colony formation assays both indicated that silencing SOX7 in miR-184 inhibitor transfected cells increased the proliferation of cells (Figure 5, B and C, Figure S4, B and C). The anchorage-independent growth assay showed similar results (Figure 5D, Figure S4D). These results suggested that silencing SOX7 expression in miR-184-repressed cells could reverse the inhibitory effect of the miR-184 inhibitor on HCC cell proliferation. Consistently, the growth rate of miR-184-inhibited cells was further inhibited by SOX7 overexpresion (Figure S5). These data confirmed that miR-184 promotes HCC cell proliferation and tumorigenicity by repressing endogenous SOX7 expression, and that SOX7 suppression is essential for miR-184-mediated HCC cell proliferation.

## Discussion

**Figure 6:**
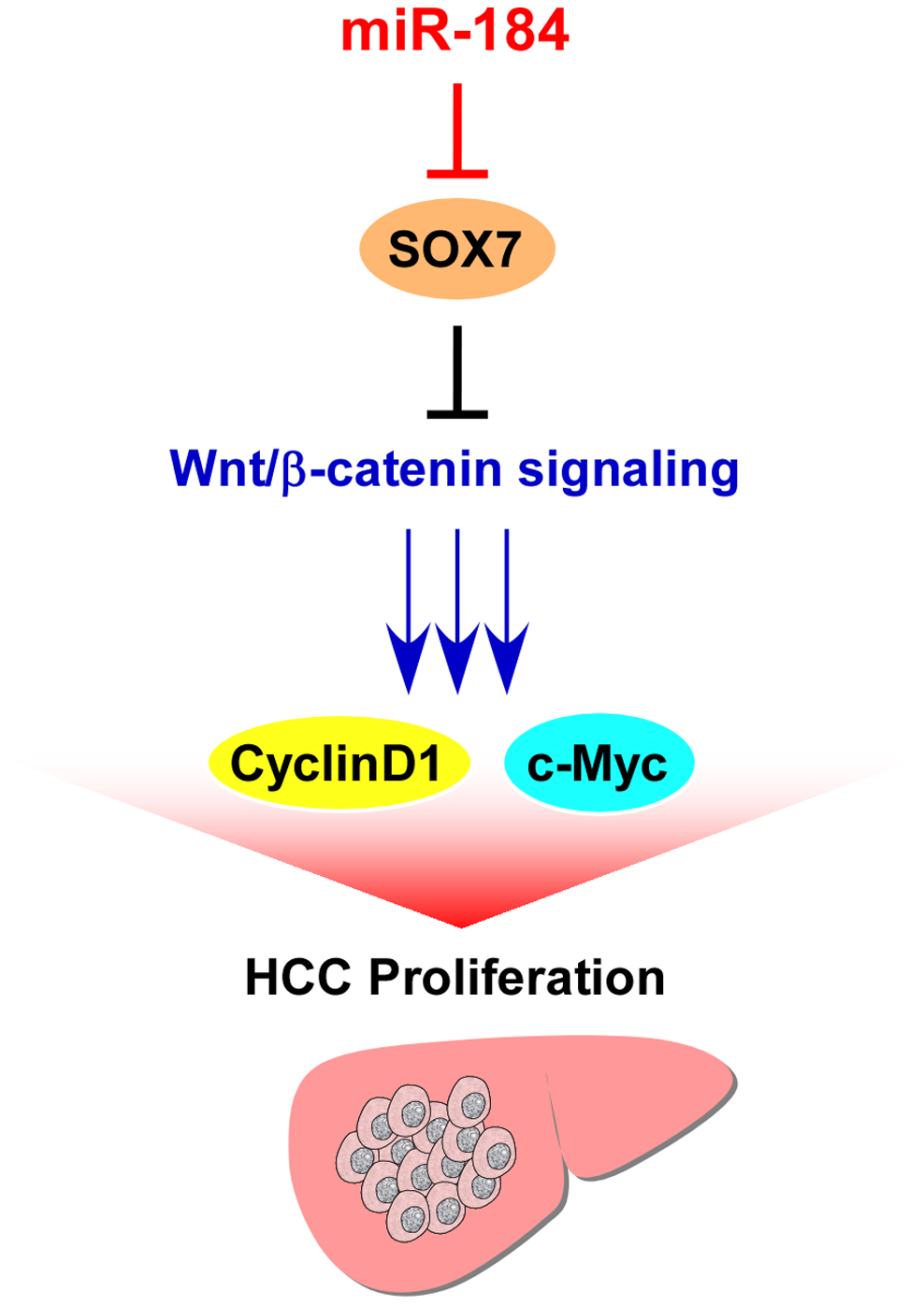
Proposed model. Upregulation of miR-184 represses SOX7 and promotes cell proliferation in HCC.

In the present study, we found that miR-184 is upregulated in HCC cell lines and tissues. Ectopic expression of miR-184 promotes the proliferation and tumorigenicity of HCC cells by targeting the 3′-UTR of SOX7 mRNA and suppressing its expression. The negative regulation of SOX7 by miR-184 leads to upregulation of c-Myc, CyclinD1 and Rb phosphorylation (Figure 6). We demonstrated that miR-184 might have an important role in the SOX7-mediated signal pathway during HCC progression.

Accumulating evidence suggests that aberrant expression of miRNAs in tumors contributes to human tumorigenesis by affecting the expression of multiple genes [10], [13], [29]. Some studies have suggested miR-184 as a potential onco-miR. One comprehensive microRNA profiling of prostate cancer showed that miR-184 was upregulated in high grade tumors [30]. Mature miR-184 was also found to be upregulated in squamous cell carcinoma of the tongue. Inhibition of miR-184 in tongue SCC cells reduced cell proliferation and induced apoptosis [31]. Xu *et al.* found that the TNFAIP2 miR-184 binding site variant rs8126 T>C genotype was significantly associated with risk of gastric cancer development [32]. Other studies showed that miR-184 has a tumor-suppressive role in cancers. MiR-184 inhibits neuroblastoma cell survival and promotes apoptosis by targeting AKT2 [33]. Another study demonstrated that miR-184 dramatically reduces tumor growth and increases overall survival in an orthotopic murine model of neuroblastoma [34]. Taken together, these studies indicate a possible role of miR-184 in modulating tumor progression. Recently, Gao *et al.* reported that miR-184 functions as an oncogenic regulator in HCC. They found miR-184 might play a role in proliferation of HCC cells by regulating INPPL1 expression and act as anti-apoptotic factor in HCC development by inhibiting the activities of caspases 3/7 [35]. However, the biological function and mechanism of miR-184 in HCC has not been completely elucidated. Our study revealed that miR-184 is upregulated in HCC and promotes HCC cell proliferation *in vitro* and suggested an oncogenic role of miR-184 in HCC and identify a possible target for HCC diagnosis and therapy. The proliferative function of miR-184 in HCC might be through SOX7-mediated signaling pathway regulation, such as Wnt/β-catenin pathway. Nevertheless, the expression level of miR-184 in HCC and its clinical relevance, require further study.

Among the putative targets of miR-184 in the publicly available algorithms, SOX7 is reported to have a tumor-suppressive function in tumors [19]–[24]. SOX7 was reported to be downregulated in lung cancer and forced-expression of SOX7 reduces cell proliferation, increases sub-G1 phase of cell cycle and increases apoptosis of non-small cell lung cancer (NSCLC) cells [19]. Li *et al*. found that decreased expression of SOX7 is correlated with poor prognosis in lung adenocarcinoma patients [20]. Furthermore, Chan *et al*. reported that SOX7 was significantly downregulated in high grade endometrial cancer, and overexpression of SOX7 reduced endometrial cancer cell growth via suppression of Wnt/β-catenin signaling [21]. It has been demonstrated that SOX transcription factors are DNA-binding HMG domain proteins. They regulate some of the same processes as the Wnt/β-catenin signaling pathway, including tissue specification, organ development, stem cell homeostasis and cancer progression [27], [36]. As a member of the SOXF subfamily, SOX7 is reported to be involved in the modulation of the Wnt/β-catenin signaling pathway, significantly reducing Wnt/β-catenin-stimulated transcription, through a motif that enables SOX7 to bind to β-catenin [28]. Considering the importance of Wnt/β-catenin signaling pathway in tumorigenesis and the regulatory role of SOX7 in this signaling pathway, whether miR-184 performs its function in HCC via SOX7/Wnt/β-catenin signaling pathway requires further investigation.

In summary, our findings demonstrate, for the first time, that miR-184 plays an important role in HCC development and progression. The results reveal that miR-184 is overexpressed in HCC and overexpression of miR-184 promotes cell proliferation and tumorigenesis in human HCC cells. Furthermore, we identified SOX7 mRNA as a direct and functional target of miR-184. In addition, SOX7 suppression is essential for miR-184-induced cell proliferation in HCC. Further study is required to identify the biological function of miR-184 and its clinical relevance in HCC development. Collectively, we believe that miR-184 plays an essential role in the progression of HCC and might represent a therapeutic target for HCC.

## Supporting Information

Figure S1 **The expression of miR-184 was examined in eight paired cancerous tissues (T) and their adjacent noncancerous hepatic tissues (ANT).** The average miR-184 expression was normalized using U6 expression. Error bars represent the mean ± SD from three independent experiments. *P<0.05.
(TIF)

Figure S2 **The proliferative capacity and SOX7 expresion of THLE3. A**. The proliferative capacity of THLE3 cells and THLE3 cells transfected with miR-184 inhibitor, compared with HCC cells, Hep3B and Huh7, analyzed by the MTT assay. **B**. The expression levels of SOX7 protein in THLE3 cells, Hep3B and Hep3B transfected with miR-184 inhibitor cells, Huh7 and Huh7 transfected with miR-184 inhibitor cells.
(TIF)

Figure S3 **The expression of miR-184, and Wnt/β-catenin signaling related genes in HCC tissues.** **A**. Real-time PCR analysis of *miR-184, Cyclin D1, MYC* and *LEF1* in HCC tissues. **B**. The correlation between miR-184 expression and *Cyclin D1, MYC* or *LEF1* expression in HCC tissues. Error bars represent the mean ± SD from three independent experiments, *P*<0.05.
(TIF)

Figure S4 **MiR-184 promotes HCC proliferation by inhibiting SOX7. A**. The expression levels of SOX7 in miR-184-inhibitor transfected HCC cells that were transfected with SOX7-siRNA#2, as measured by western blotting; α-Tubulin served as the loading control. **B**. The growth rates in SOX7-silenced cells, as indicated by the MTT assay. **C**. Quantifications of crystal violet stained cell colonies formed by indicated HCC cell lines, 10 days after inoculation. **D**. Quantifications of colony numbers of indicated cells determined by an anchorage-independent growth assay. Colonies larger than 0.1 mm in diameter were scored. Error bars represent the mean ± SD from three independent experiments. *P<0.05.
(TIF)

Figure S5 **HCC proliferation is markedly promoted by miR-184 inhibition and SOX7 upregulation. A**. The expression levels of SOX7 in miR-184-inhibitor transfected HCC cells that overexpressing SOX7, as measured by western blotting; α-Tubulin served as the loading control. **B**. The growth rates in miR-184-inhibited and SOX7-overexpressing cells, as indicated by the MTT assay. **C**. Quantifications of crystal violet stained cell colonies formed by indicated HCC cell lines, 10 days after inoculation. **D**. Quantifications of colony numbers of indicated cells determined by an anchorage-independent growth assay. Colonies larger than 0.1 mm in diameter were scored. Error bars represent the mean ± SD from three independent experiments. *P<0.05.
(TIF)

## References

[1] Willatt JM, Francis IR, Novelli PM, Vellody R, Pandya A, et al (2012) Interventional therapies for hepatocellular carcinoma. Cancer Imaging 12: 79–88. 22487698 10.1102/1470-7330.2012.0011 PMC3335329

[2] Jemal A, Bray F (2011) Center MM, Ferlay J, Ward E, et al (2011) Global cancer statistics. CA Cancer J Clin 61: 69–90. 21296855 10.3322/caac.20107

[3] Lei J, Yan L (2012) Comparison between living donor liver transplantation recipients who met the Milan and UCSF criteria after successful downstaging therapies. J Gastrointest Surg 16: 2120–2125. 22948843 10.1007/s11605-012-2019-y

[4] Takayama T (2011) Surgical treatment for hepatocellular carcinoma. Jpn J Clin Oncol 41: 447–454. 21411469 10.1093/jjco/hyr016

[5] Ambros V (2004) The functions of animal microRNAs. Nature 431: 350–355. 15372042 10.1038/nature02871

[6] Bartel DP (2004) MicroRNAs: genomics, biogenesis, mechanism, and function. Cell 116: 281–297. 14744438 10.1016/s0092-8674(04)00045-5

[7] Jiang L, Lin C, Song L, Wu J, Chen B, et al (2012) MicroRNA-30e* promotes human glioma cell invasiveness in an orthotopic xenotransplantation model by disrupting the NF-kappaB/IkappaBalpha negative feedback loop. J Clin Invest 122: 33–47. 22156201 10.1172/JCI58849 PMC3248293

[8] Hagan JP, Croce CM (2007) MicroRNAs in carcinogenesis. Cytogenet Genome Res 118: 252–259. 18000378 10.1159/000108308

[9] Calin GA, Croce CM (2006) MicroRNA signatures in human cancers. Nat Rev Cancer 6: 857–866. 17060945 10.1038/nrc1997

[10] Farazi TA, Hoell JI, Morozov P, Tuschl T (2013) MicroRNAs in human cancer. Adv Exp Med Biol 774: 1–20. 23377965 10.1007/978-94-007-5590-1_1 PMC3704221

[11] Kusenda B, Mraz M, Mayer J, Pospisilova S (2006) MicroRNA biogenesis, functionality and cancer relevance. Biomed Pap Med Fac Univ Palacky Olomouc Czech Repub 150: 205–215. 17426780 10.5507/bp.2006.029

[12] Markou A, Liang Y, Lianidou E (2011) Prognostic, therapeutic and diagnostic potential of microRNAs in non-small cell lung cancer. Clin Chem Lab Med 49: 1591–1603. 21767219 10.1515/CCLM.2011.661

[13] Cho WC (2007) OncomiRs: the discovery and progress of microRNAs in cancers. Mol Cancer 6: 60. 17894887 10.1186/1476-4598-6-60 PMC2098778

[14] Gandillet A, Serrano AG, Pearson S, Lie ALM, Lacaud G, et al (2009) Sox7-sustained expression alters the balance between proliferation and differentiation of hematopoietic progenitors at the onset of blood specification. Blood 114: 4813–4822. 19801444 10.1182/blood-2009-06-226290

[15] Cermenati S, Moleri S, Cimbro S, Corti P, Del Giacco L, et al (2008) Sox18 and Sox7 play redundant roles in vascular development. Blood 111: 2657–2666. 18094332 10.1182/blood-2007-07-100412

[16] Francois M, Koopman P, Beltrame M (2010) SoxF genes: Key players in the development of the cardio-vascular system. Int J Biochem Cell Biol 42: 445–448. 19733255 10.1016/j.biocel.2009.08.017

[17] Seguin CA, Draper JS, Nagy A, Rossant J (2008) Establishment of endoderm progenitors by SOX transcription factor expression in human embryonic stem cells. Cell Stem Cell 3: 182–195. 18682240 10.1016/j.stem.2008.06.018

[18] Savage J, Conley AJ, Blais A, Skerjanc IS (2009) SOX15 and SOX7 differentially regulate the myogenic program in P19 cells. Stem Cells 27: 1231–1243. 19489079 10.1002/stem.57

[19] Hayano T, Garg M, Yin D, Sudo M, Kawamata N, et al (2013) SOX7 is down-regulated in lung cancer. J Exp Clin Cancer Res 32: 17. 23557216 10.1186/1756-9966-32-17 PMC3648366

[20] Li B, Ge Z, Song S, Zhang S, Yan H, et al (2012) Decreased expression of SOX7 is correlated with poor prognosis in lung adenocarcinoma patients. Pathol Oncol Res 18: 1039–1045. 22777918 10.1007/s12253-012-9542-8

[21] Chan DW, Mak CS, Leung TH, Chan KK, Ngan HY (2012) Down-regulation of Sox7 is associated with aberrant activation of Wnt/b-catenin signaling in endometrial cancer. Oncotarget 3: 1546–1556. 23295859 10.18632/oncotarget.667 PMC3681493

[22] Zhang Y, Huang S, Dong W, Li L, Feng Y, et al (2009) SOX7, down-regulated in colorectal cancer, induces apoptosis and inhibits proliferation of colorectal cancer cells. Cancer Lett 277: 29–37. 19108950 10.1016/j.canlet.2008.11.014

[23] Guo L, Zhong D, Lau S, Liu X, Dong XY, et al (2008) Sox7 Is an independent checkpoint for beta-catenin function in prostate and colon epithelial cells. Mol Cancer Res 6: 1421–1430. 18819930 10.1158/1541-7786.MCR-07-2175 PMC2652859

[24] Katoh M (2002) Expression of human SOX7 in normal tissues and tumors. Int J Mol Med 9: 363–368. 11891528

[25] Hahn WC, Dessain SK, Brooks MW, King JE, Elenbaas B, et al (2002) Enumeration of the simian virus 40 early region elements necessary for human cell transformation. Mol Cell Biol 22: 2111–2123. 11884599 10.1128/MCB.22.7.2111-2123.2002 PMC133688

[26] Li J, Zhang N, Song LB, Liao WT, Jiang LL, et al (2008) Astrocyte elevated gene-1 is a novel prognostic marker for breast cancer progression and overall patient survival. Clin Cancer Res 14: 3319–3326. 18519759 10.1158/1078-0432.CCR-07-4054

[27] Kormish JD, Sinner D, Zorn AM (2010) Interactions between SOX factors and Wnt/beta-catenin signaling in development and disease. Dev Dyn 239: 56–68. 19655378 10.1002/dvdy.22046 PMC3269784

[28] Takash W, Canizares J, Bonneaud N, Poulat F, Mattei MG, et al (2001) SOX7 transcription factor: sequence, chromosomal localisation, expression, transactivation and interference with Wnt signalling. Nucleic Acids Res 29: 4274–4283. 11691915 10.1093/nar/29.21.4274 PMC60197

[29] Esquela-Kerscher A, Slack FJ (2006) Oncomirs - microRNAs with a role in cancer. Nat Rev Cancer 6: 259–269. 16557279 10.1038/nrc1840

[30] Walter BA, Valera VA, Pinto PA, Merino MJ (2013) Comprehensive microRNA Profiling of Prostate Cancer. J Cancer 4: 350–357. 23781281 10.7150/jca.6394 PMC3677622

[31] Wong TS, Liu XB, Wong BY, Ng RW, Yuen AP, et al (2008) Mature miR-184 as Potential Oncogenic microRNA of Squamous Cell Carcinoma of Tongue. Clin Cancer Res 14: 2588–2592. 18451220 10.1158/1078-0432.CCR-07-0666

[32] Xu Y, Ma H, Yu H, Liu Z, Wang LE, et al (2013) The miR-184 binding-site rs8126 T>C polymorphism in TNFAIP2 is associated with risk of gastric cancer. PLoS One 8: e64973. 23724109 10.1371/journal.pone.0064973 PMC3665554

[33] Foley NH, Bray IM, Tivnan A, Bryan K, Murphy DM, et al (2010) MicroRNA-184 inhibits neuroblastoma cell survival through targeting the serine/threonine kinase AKT2. Mol Cancer 9: 83. 20409325 10.1186/1476-4598-9-83 PMC2864218

[34] Tivnan A, Foley NH, Tracey L, Davidoff AM, Stallings RL (2010) MicroRNA-184-mediated inhibition of tumour growth in an orthotopic murine model of neuroblastoma. Anticancer Res 30: 4391–4395. 21115884 PMC5030819

[35] Gao B, Gao K, Li L, Huang Z, Lin L (2013) miR-184 functions as an oncogenic regulator in hepatocellular carcinoma (HCC) Biomed Pharmacother. In press. 10.1016/j.biopha.2013.09.005 24183204

[36] Katoh Y, Katoh M (2005) Comparative genomics on SOX2 orthologs. Oncol Rep 14: 797–800. 16077994

