# Supplementary figures and images for: Mir-184 Post-Transcriptionally Regulates SOX7 Expression and Promotes Cell Proliferation in Human Hepatocellular Carcinoma
Source: PLoS One. 2014 Feb 18;9(2):e88796. doi: 10.1371/journal.pone.0088796 (PMC3928300; doi:10.1371/journal.pone.0088796)

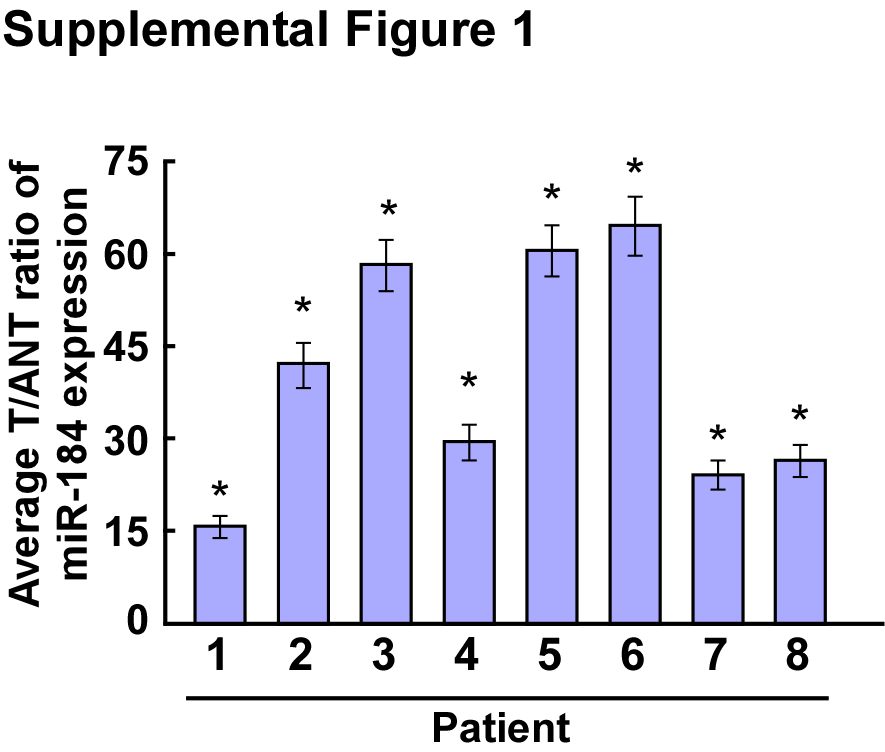

Supplement: Figure S1 — The expression of miR-184 was examined in eight paired cancerous tissues (T) and their adjacent noncancerous hepatic tissues (ANT). The average miR-184 expression was normalized using U6 expression. Error bars represent the mean ± SD from three independent experiments. *P<0.05. (TIF) [file pone.0088796.s001.tif]

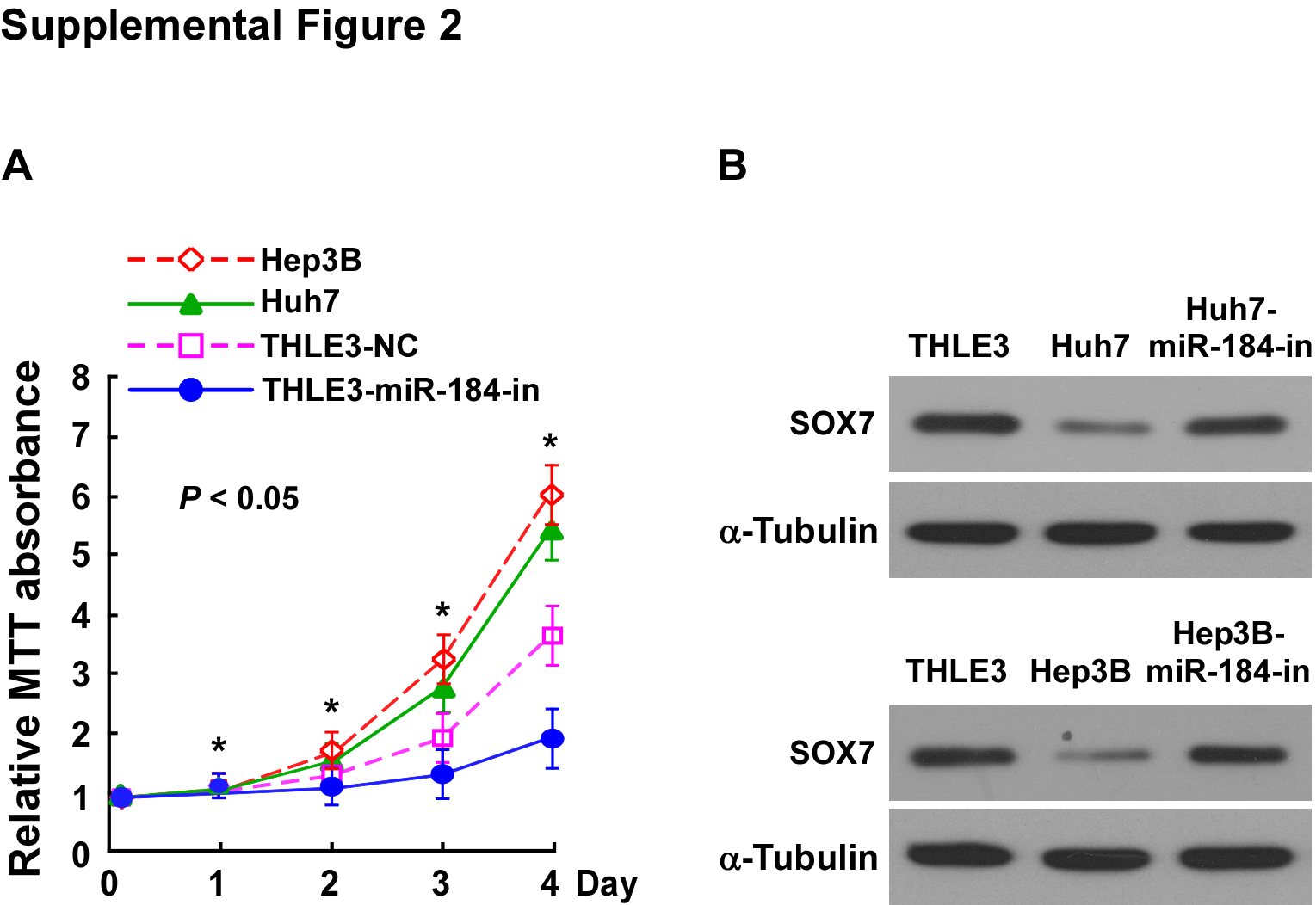

Supplement: Figure S2 — The proliferative capacity and SOX7 expresion of THLE3. A. The proliferative capacity of THLE3 cells and THLE3 cells transfected with miR-184 inhibitor, compared with HCC cells, Hep3B and Huh7, analyzed by the MTT assay. B. The expression levels of SOX7 protein in THLE3 cells, Hep3B and Hep3B transfected with miR-184 inhibitor cells, Huh7 and Huh7 transfected with miR-184 inhibitor cells. (TIF) [file pone.0088796.s002.tif]

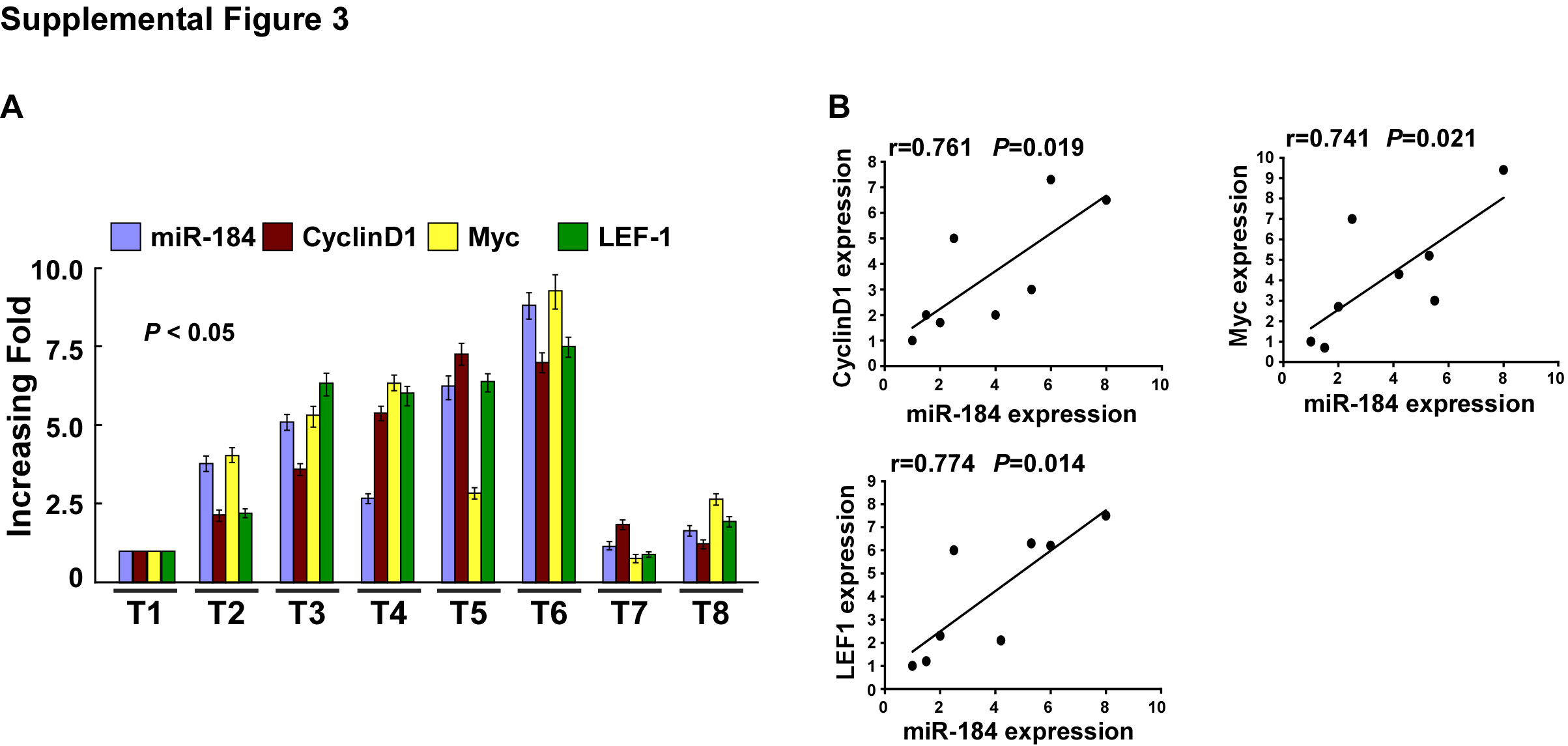

Supplement: Figure S3 — The expression of miR-184, and Wnt/β-catenin signaling related genes in HCC tissues. A. Real-time PCR analysis of miR-184, Cyclin D1, MYC and LEF1 in HCC tissues. B. The correlation between miR-184 expression and Cyclin D1, MYC or LEF1 expression in HCC tissues. Error bars represent the mean ± SD from three independent experiments, P<0.05. (TIF) [file pone.0088796.s003.tif]

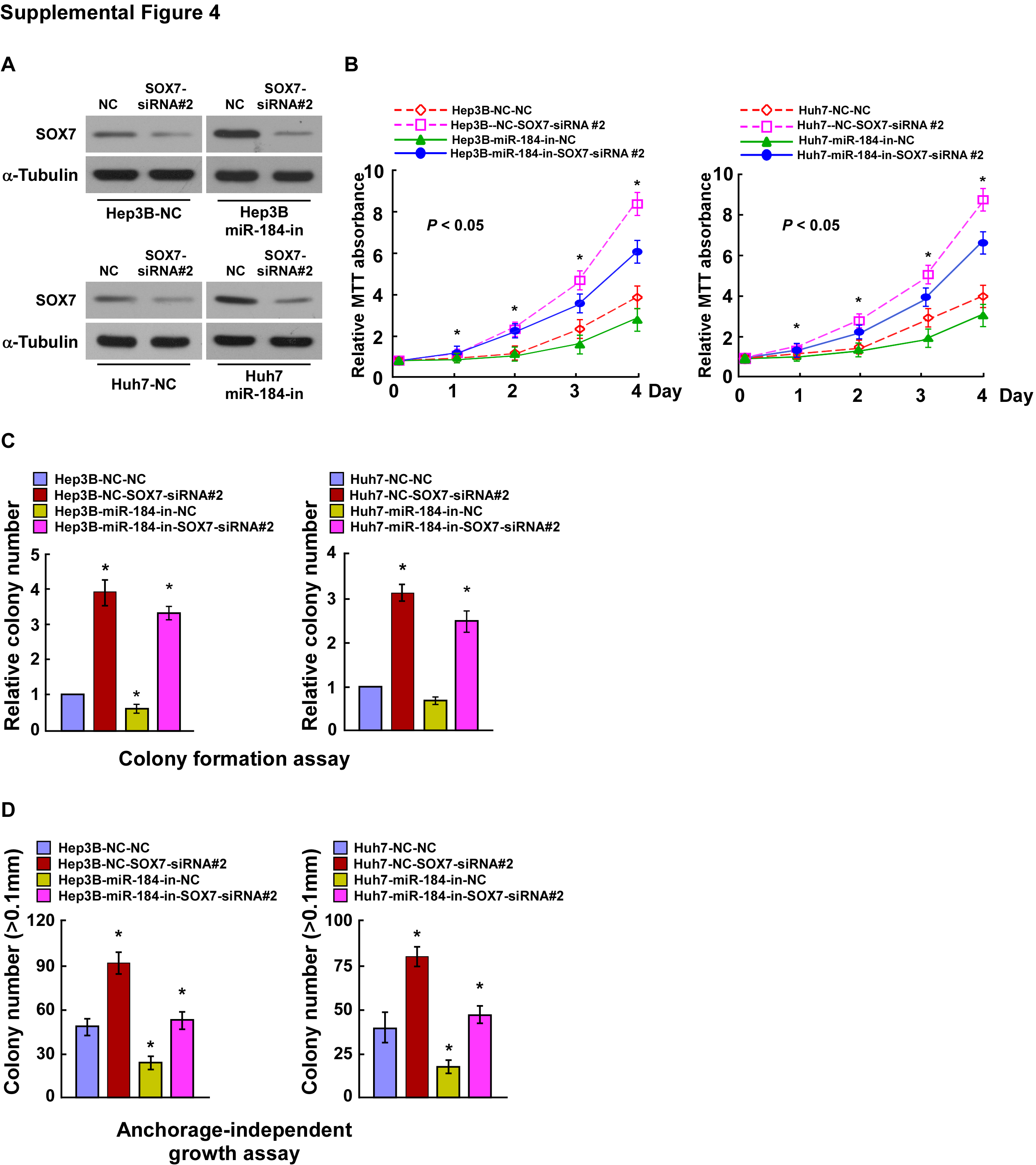

Supplement: Figure S4 — MiR-184 promotes HCC proliferation by inhibiting SOX7. A. The expression levels of SOX7 in miR-184-inhibitor transfected HCC cells that were transfected with SOX7-siRNA#2, as measured by western blotting; α-Tubulin served as the loading control. B. The growth rates in SOX7-silenced cells, as indicated by the MTT assay. C. Quantifications of crystal violet stained cell colonies formed by indicated HCC cell lines, 10 days after inoculation. D. Quantifications of colony numbers of indicated cells determined by an anchorage-independent growth assay. Colonies larger than 0.1 mm in diameter were scored. Error bars represent the mean ± SD from three independent experiments. *P<0.05. (TIF) [file pone.0088796.s004.tif]

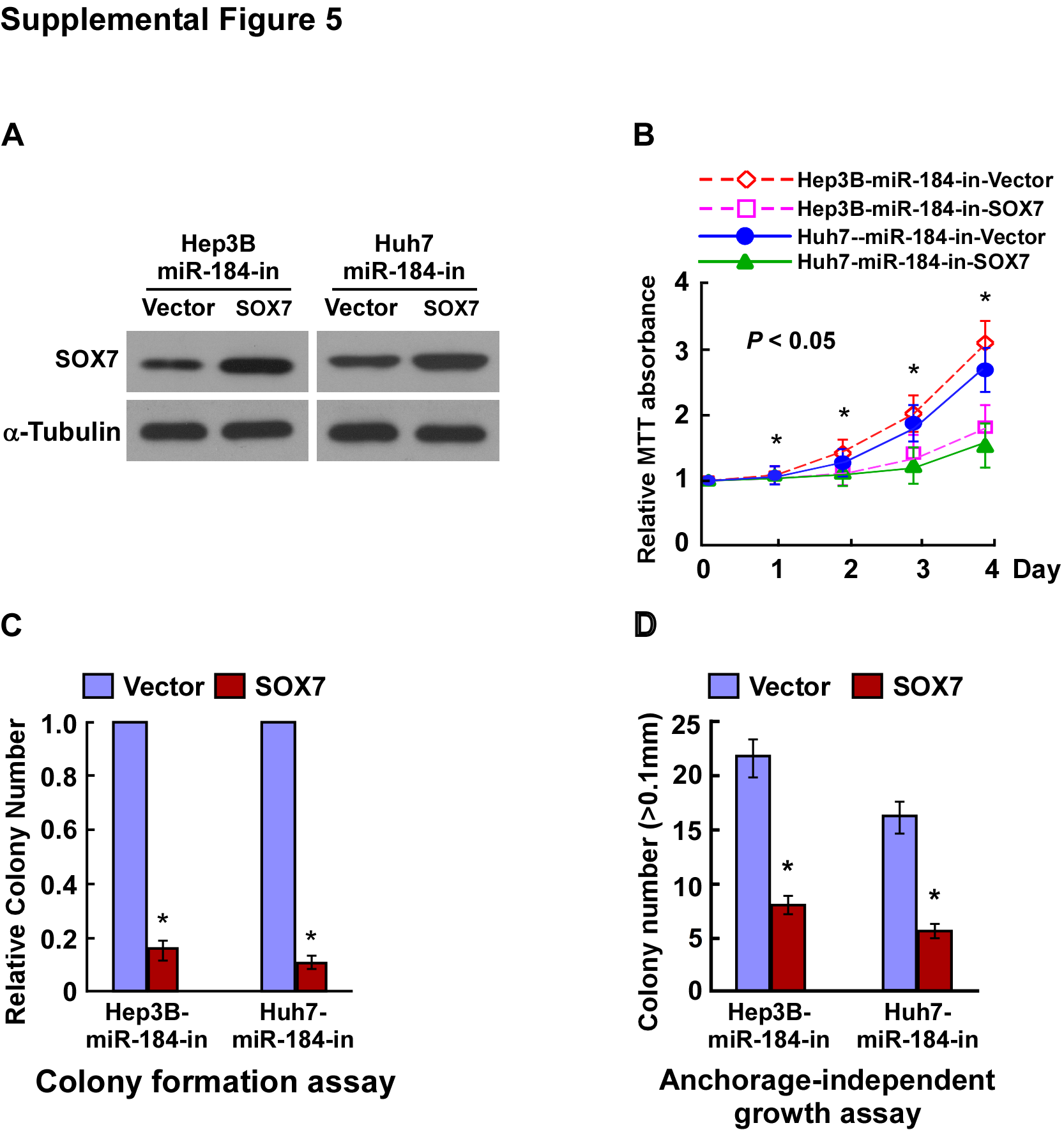

Supplement: Figure S5 — HCC proliferation is markedly promoted by miR-184 inhibition and SOX7 upregulation. A. The expression levels of SOX7 in miR-184-inhibitor transfected HCC cells that overexpressing SOX7, as measured by western blotting; α-Tubulin served as the loading control. B. The growth rates in miR-184-inhibited and SOX7-overexpressing cells, as indicated by the MTT assay. C. Quantifications of crystal violet stained cell colonies formed by indicated HCC cell lines, 10 days after inoculation. D. Quantifications of colony numbers of indicated cells determined by an anchorage-independent growth assay. Colonies larger than 0.1 mm in diameter were scored. Error bars represent the mean ± SD from three independent experiments. *P<0.05. (TIF) [file pone.0088796.s005.tif]
